# Proceedings of British Dental Association

**Published:** 1884-11

**Authors:** 


AMERICAN JOURNAL

OF

DENTAL SCIENCE.

Vol. xviii. THIRD SERIES—NOVEMBER, 1884. No. 7.

ARTICLE I.

### BRITISH DENTAL ASSOCIATION.

### Western Branch.

The Annual Meeting of this Branch was held at Torquay, on August 2nd Owing to the absence of the retiring President (Mr. Charles Gaine), the President-elect (Mr. W. A. Hunt) occupied the chair.

It was decided to hold the next Annual Meeting in Hereford, and that Mr. McAdam be President-elect.

A question arose as to the eligibility of Mr. Washbourne to a place on the Council of the branch, on account of his publishing the following advertisement :—

Mr. Norman Washbourne, Bridge House, Taunton, visits Minehead on the third Monday in each month, at Mr. Floyd's 2, Wellington Square. The next visit will be on August 18th. He also visits Williton on the second Monday in each month, at the Post Office, Mr. Hales' his first visit being on September 8th. Hours 10 to 4. Appointments can be made by writing previously to Bridge House, Taunton."

Mr. Browne-Mason said this was not an advertisement that could be objectionable, from the simple fact that the gentleman did not even mention his profession. It seemed to him an announcement that was justifiable ; but seeing Mr. Smith-Turner present, he should like to hear what he had to say on the subject, as his was an opinion that would have much weight.

Mr. Smith-Turner said the answer to this question was not to be given in a few words. They knew that the object of this Association was to raise not only the social status, but the moral tone of their profession, and one of their great purposes was to discourage a certain mode of advertising which was quite unprofessional. Any body of men claiming to be professional men must be prepared to give up what might be regarded as a lucrative practice, if it was necessary that they should do so, to secure a higher social status. The system of advertising generally pursued was of a very objectionable character, but it seemed to him that an announcement of the kind just read could not be prohibited by any Association without seriously interfering with what might be termed the liberty of the subject. This gentleman did not say what was his profession, or what was the object of his visit, but simply stated that he would visit a certain place at a certain time. It was simply a personal announcement by the gentleman for the convenience of his patients and the public generally. He could not see that it affected his professional position in any way, or his right to be appointed to the Council, or the wisdom of the Council in nominating him. If there were no advertisements of any other kind than these to trouble them their position would be a very happy one indeed. He did not look upon this advertisement as having any objectionable feature, and did not think the Odontological Society would interfere with it.

The President said he was very glad indeed to find that his own opinion coincided with that of Mr. Smith-Turner. He had been asked his opinion on the matter, and had given it in very similar terms. Now, although the gentleman in question might be perfectly justified in publishing this announcement, yet he thought that it was a course the members at large would rather not see adopted. He was glad to say that he believed that if a little influence were used these announcements would not be seen in the future.

Mr. Smith-Turner thought it said a great deal for the Association that it had been able in so short a time to infuse so wholesome a spirit in the profession. They must not forget what they had been in their efforts to strive to make themselves something different.

The President (Mr. Hunt), in his inaugural address, considered the dental profession a scientific profession, undergoing healthy consolidation from day to day.

Dental surgery has with us, until very lately, been passing through its embryonic condition, and yet it needs much careful attention ; but in spite of advertisements steeped in falsehood, the genuine services of the educated and competent dental surgeon are more than ever in request, and will continue to be so as civilization advances, and as the true and close relationship of sound teeth to health and enjoyment is better understood. Many of the false lights of quackery will go smokily out ; and the true light will be more clearly recognized, and more unhesitatingly followed.

So, then there is every reason why we should march forward hand in hand with the spirit of progress and the true science.

But it has been the nature of scientific truth in the past to unfold herself slowly, rather than by leaps and bounds. She needed diligent inquiry before she yielded herself up. Indeed, as a rule, the beginnings of science are not sudden and palpable, but slow and insensible. Mr. Hunt then referred to the works of John Hunter, published in 1778, of Joseph Fox, of Thomas Bell, of Nasmyth, and of (now) Sir Richard Owen, K. C. B. He dwelt upon these points to demonstrate, by means of the dates given, the slow and unfolding of the truth in the past. As relates to our calling, antiquity has given us nothing, except what to avoid. Now the torch seems fully ablaze, and the world has been further enriched by the publication of the works of Messrs. Tomes (father and son) and some other valuable publications which will occur to us all. Moreover, "a wave of progress" has reached this country from the other side of the Atlantic which has had its stimulus upon us, and has raised an increasing demand for, and appreciation of our services.

To education, broad as well as technical, Mr. Hunt looks for the elimination of the false and worthless from our ranks, rather than to all the acts of Parliament that have been or ever will be. Next, therefore, to compulsory registration, let us rank the Preliminary Examinations in the subjects of an English gentleman's education, a knowledge of which no man who has any wish to secure social recognition can afford to be without, and which is now happily compulsory on all who shall henceforth enter our ranks.

In truth, he continued, the current of knowledge and improvement rushes on so swiftly that those who hesitate to commit themselves to it will soon be left far behind, and serve only the ignoble purpose of enabling us to measure the force and rapidity of the stream. Our own individual credit, and the honor and reputation of our body, demand that we should not be wholly ignorant of the other departments of the healing art, and in general science, history, antiquities, music, the fine arts, &c., as our individual tastes prompt us, we should be students.

Let us each, then, strain every nerve, not only to advance the branch of the profession we have chosen, but to advance our own individual culture as much as we can ; thus shall we enjoy the greatest pleasure which upright and honorable minds can conceive—that of increasing the *usefulness*, and thereby raising the credit and respectability, of the body to which we belong, and so prepare for ourselves, at all times, a pure source of the most satisfactory reflections.

Mr. W. V. Moore then read a paper on "The Condition of the Tonsils Affecting the Teeth." The gist of the argument was that an unhealthy condition of the tonsils often promotes caries, and also pain and inflammation of the gums. Mr. Moore mentioned cases which had come under his notice at the Plymouth Dispensary, and stated the remedies he had employed.

Mr. Pearman showed two germinated temporary teeth the right upper central and lateral, which he had extracted from the mouth of a little girl six and a-half years old.

Mr. Balkwill related the particulars of a casc of transplantation. In May last a young lady had her first upper right bicuspid taken out because there was not room for the canine. Her mother said, " what a pity it is to take it out —it's not a bit decayed." He said " yes," and added that if her sister had a corresponding tooth which was decayed it would be well to change it. Curiously it was a fact that the corresponding tooth in the sister had been troubling her for some little time, and on looking at the upper bicuspid in thc sister's mouth he discovered that although there was not acually a fistula there was a red place. Under gas he took out the decayed tooth from the elder sister, inserted in its place the sound tooth he had taken from the younger sister, pressed it up and kept it firmly there with his finger for five minutes (he preferred this to any attachment, or plate, or tying it afterwards) told her to keep her teeth tight shut for a time and be careful in eating for the next few days. He saw the case a week later; it was a little tender but doing well. He saw it a fortnight since when it was almost as tight as the other teeth. The threatened alveolar abscess had completely disappeared, any everything was looking as healthy and satisfactory as it could be.

The President remarked that there were numberless cases on record of teeth accidentally knocked out being replaced, not by an educated dentist even, but by bystanders, and there had been actual re-union of the vessels, pulp and nerves. When a tooth was taken out it did not at once die, the vessels had vitality for some hours afterwards. He did not think there was any necessity to remove the pulp from a living tooth which had lost its connection with its socket.

Mr. Keys said the cases mentioned by the President were of teeth restored to the places from which they had been removed—they were different from the case of a tooth taken out of one mouth and placed in another. He did not think the cases analogous, because where there was a transfer they would never get a perfect fit.

The President said his remarks applied only to healthy teeth. As to the point just raised by Mr. Keys, of course the cases he had mentioned were not quite analogous to the one under discussion, but it must be remembered that not only would teeth grow when transplanted from one mouth to another, but, as was well-known, there was the instance of teeth being transplanted to the comb of a cock where they grew and thrived. The whole question was a very interesting one, and he regretted that there was not time to discuss it further.

Mr. Keys presented a model showing the plan he adopted for protecting pivot holes in plaster models. He had had trouble in putting pivots into gold plates, and the plan he adopted got over the difficulty. The device which he illustrated, consisted of placing a zinc tube over the wires in the Stent's impression, and the tubes being left in the plaster cast protection was given throughout the entire process. The idea, as illustrated by the models, was generally approved of.

Mr. Brown-Mason pivoted a front tooth in the manner referred to as Mr. Sheffield's. Subsequently Mr. Verrier gave his demonstration of continuous gum work, and Mr. Briggs showed Dr. Campbell's vulcanizer and celluloid apparatus.

In the evening the members dined together at the Imperial Hotel.

### ASSOCIATION MEETING.

The Fourth Annual General Meeting of the British Dental Association was held in Edinburgh, on August, 28th, 29 and 30th, the Royal College of Surgeons of Edinburgh having kindly granted the use of their Library and Hall for the purposes of the meeting.

The proceedings began with a reception in the Museum of the College, by Dr. John Smith, President of the College of Surgeons, and by the Lord Provost of Edinburgh, Sir George Harrison, LL. D. After luncheon, which was served in an ante-room, demonstrations and exhibits of instruments, &c,, engaged the attention of members ; and in the evening there was a gathering of members and their friends at the Forestry Exhibition.

The second day was devoted to the more important business, commencing with a meeting of the Representative Board, at 9 A. M. Demonstrations also began at the same hour. At 10.30, the General Meeting was begun, Mr. J. Smith-Turner, Vice-President, occupying the chair in the absence of the retiring President, Mr. C. Spencer-Bate, F. R. S.

Mr. John Tomes, F. R. S., President of the Representative Board, had prepared an address which was read at the meeting of the Board, and it was decided that it be also read to the general assembly.

In referring to the lately-withdrawn Medical Bill, Mr. Tomes remarked that the dental section was of advantageous importance to them. One result of its passing would have been the repeal of a provision introduced in the Dentist's Act rendering the consent of the Medical Council a necessary antecedent to a prosecution under section 4 of the Act. The question of direct representation in the Medical Council, so much agitated for by medical practitioners, and in the bill to some extent conceded, was, as respects dental practitioners, brought before the Royal Commission and considered with a result that, taking into account the very recent legal recognition of the dental profession, as such, might have foreseen. While, as the decision of the Royal Commission would indicate, the Licentiates were for the present sufficiently represented, those who held no registrable educational qualification were not unrepresented. Legislation would not, indeed could not, afford a remedy for this evil, but education and association will tend to its diminution; and the increasing prosperity of their dental schools, brought into action by legislation, bade them hope that a professional nuisance which never could be wholly removed would be brought into much narrower limits, and its practice confined to the few meaner spirits whose presence in their ranks might be regretted but could not be prevented. Those who lived on might see, and he would venture to predict would see that, happen what might, their Act if rightly used by themselves, was sufficient, and that the proposed amendments, though desirable, were of relatively small account as respects the general results. No fault had been found with the strictly educational clauses or with the manner in which effect had been given to them by the Medical Council, and therein lay the strength of their social and professional position. If full force were given to these, they might regard lightly the mere prohibitory sections of the Act. Education was the highest form of protection. This secured, its possessor might defy the charlatan whom many contend should be put down by the strong hand of the law. The government steadily refused to make the act of medical practice by unqualified and unregistered persons penal. The most that could be obtained was the prevention of their use of the title of the qualified, their establishment of a claim for for pecuniary remuneration for attendance, or their appointment to any medical office whatever. And with this the medical profession and its dental branch must be content.

Mr. Hutchinson, in the place of Mr. Oakley Coles, read a report of the Dental Benevolent Fund Committee. It was stated that it was in contemplation of the Dental Hospital of London and of the National Dental Hospital to found Scholarships to enable boys who turn out good students to receive free education at these dental hospitals. also that Sir Edwin Saunders, as a trustee, had been instructed to invest the sum of £500, and that he had done so in 2½ per cent. Consols, the sum amounting now to £535 8s. 9d. The report was adopted.

Mr. Parkinson, the Treasurer, stated that their balance in bank amounted to £528. He was sorry to add that they had at the same time 135 unpaid subscriptions for one year, and two years there were thirty subscriptions paid. Mr. Canton, the Hon. Secretary, submitted the fifth annual report, which stated that there was an increase in every direction. Since the last meeting at Plymouth there had been one prosecution—the Association *v.* Halford—and a

Scottish case was coming up in October. During last year another branch had been added to the Association—the West of Scotland branch, this being now the fifth branch. The membership of the Association now numbered 536, showing an increase of 42 ; and in addition to that six members were elected on the opening day to the Scottish branch, and four yesterday, making the increase 52 members. It was agreed, that the next meeting of the Association should be held in Cambridge, on August 27, 28th and 29, and Mr. White, Norwich, President of the Eastern Counties Branch, was nominated President-elect. The meeting endorsed the action of the Representative Board in removing the name of Mr. Partridge, London, from the list of members, for alleged "disreputable advertising," after remonstrances by the Board. It was reported that a Central Counties Branch was being formed, Birmingham being the centre, and that already a large number of names had been received.

The Chairman then read the President's valedictory address. Mr. Bate observed that he felt no misgivings as to the success of the meeting of the Dental Association in Edinburgh. The Association had within itself all that was necessary to make this a certainty, though the pleasure of the meeting could not fail to be enhanced by the fact of its being held in a town famed both for its literary and scientific societies, and for its poetical and historical reminiscencies. With Edinburgh were associated the names of Goodsir, Blake, Nasmyth, and several others, whose researches into the structure and mode of development of the dental tissues had rendered most important services to that branch of anatomy which formed the special study of the members of the Association.

Directing the attention of the Association to the advantages to be derived from the collection and investigation of some of the many unsolved problems still to be met with in the department of dental surgery, he remarked that the cause of decay of the teeth had been carefully studied by many, and the futility of individual research was well exemplified by the result that almost every inquirer had founde a new theory for himself, each of which was identified with the idea preconceived before he ever commenced to study the subject. It was, then, most desirable that subjects of this kind, which it was important should be set at rest for the benefit of sound curative treatment, should be investigated by two or more kinds in concert. He advocated the appointment of small committees for this purpose. Such a committee working together would, by the division of labor, be able more thoroughly to investigate what had already been done, so as to avoid the republication of observations and opinions which had long since been disproved and been forgotten ; and having thus placed themselves fairly abreast of the most reliable information, past and present, would reserve their energies for really original investigations. Amongst the subjects which might be taken in hand with advantage, besides dental caries, with the best means of preserving and utilizing the roots of teeth after the loss of their crowns by disease or accident, the extraction and replantation of teeth as a means of treating some forms of disease and restoring the teeth to usefulness. Various pathalogical conditions of the mouth also required enlarged inquiry and more extended investigation. A carefully considered report of a series of cases would be of far greater value than the scattered records of remarkable examples, which could only demonstrate the possibilities of abnormal conditions, and did little to advance the present state of our knowledge. Concluding with some remarks on the present state of dental education, Mr. Bate specially deprecated the existing inequalities in the standard required by the different examining bodies for the dental diploma. All feeling of rivalry in this respect should, he thought, be reduced to a minimum. He thought the various examining boards should come to some arrangement with the view of making the standard as uniform as possible, thus doing away with a frequent source of jealousy, and greatly strengthing the the *esprit de corps* of the profession.

The President-elect, Dr. John Smith, LL. D., President of the Royal College of Surgeons, Edinburgh, then took the chair, and delivered his Inaugural Address, He said :—" Gentlemen, on taking the chair at this large, important, and influential gathering, I need not express the sense I feel of the high honor conferred upon me by my election as President of the British Dental Association. In acknowledging the compliment thus paid me, I have to assure you that I shall do all that in me lies to discharge the duties and maintain the dignity of this distinguished office, and, so far as my abilities permit, endeavor to uphold the honorable and eminent position which the Association has already attained under my able and illustrious predecessors in this chair. The opening address by a newly-elected President at meetings such as these not unfrequently partakes of a repetitionary character—a sameness of construction and identity of topic with those of past years—a ringing of changes on by-gone addresees, necessarily engendered by the similarity of circumstances in which the successive speakers have, from time to time, been placed. Keeping this in view, and knowing that the time at our disposal in the present instance requires to be carefully economized, I shall not detain you any lengthened reiteration of what has been so often and so admirably dwelt upon before. The objects, the achievements, and the prospects of the Association are known to every one now present. I do not, therefore, propose offering any remarks upon the past, nor have I anything original to suggest in reference to the future. To enter npon the past would savor more of a valedictory than an inaugural address ; it is for the retiring President to ' cast a long and lingering look behind.' For myself, in the meantime, ' the world is all before me where to choose.' Without, then, presenting to you any prolonged eulogium upon the beneficial purposes of the British Dental Association, upon the amount of good work it has accomplished, upon the great advantages it has conferred, and the important aims and ends it has in view, towards advancing and promoting the interests of the professional body it represents, I have simply, and on all these points, only to offer you the words of unqualified congratulation that such an Association exists and flourishes. We are here to-day as the representative and supervising assembly, and, to a certain extent, as the administrative convocation for the dental profession throughout Great Britain; and being so, I have no hesitation in saying that our influence in such a capacity is felt throughout the world. It may be argued that we already have a Dental Act enunciating the laws regarding dental practice; that long before its existence we possessed at least one great dental school, and one great surgical corporation conferring on the students of that school a high and much sought-after distinctive dental qualification; that at the present day we have many additional complete and reliable schools of dentistry, and a full and recognized dental qualification conferred in each of the three divisions of the kingdom; that over all these we have a court of supervision, provided in the General Medical Council, and a safeguard and guarantee against illegal practice and pretenders in the *Dental Register;* and that, in the face of such a state of matters, little seems left for an Association of this kind to do. The answer, however, is, that by the various schemes referred to—the means and appliances—the machinery for education, examination, and qualification has been provided; but it requires some effective power to render such machinery available, and to ensure the work it is intended to perform being done, and that such work shall be done thoroughly; and, besides this, that an opportunity shall be afforded for the collective voice of the profession being heard on all the different questions it may hold to be important, that the interest and privileges of its members shall be effectively maintained, and that the laws provided for such purposes shall be enforced and carried out in their integrity. These appear to be the functions and administrative duties of an Association of this nature; and without some such representative vicarious authority as it constitutes, mere legal enactments are liable to become unexercised and of no avail.

While, however, this Association has these important duties to undertake and to pursue for the profession, it has also a duty to perform to itself. It has to keep in mind the onerous reponsibility for which it is liable officially to be held answerable in case of its actions being a failure —incurring unpalatable expenditure—or exceeding its functions or its powers. In cases of transgression, hardship, misdemeanor, what seems the proper course of action may in the abstract appear a simple matter to be put in force. But when this comes to a practical application in an exact and rigorous form, care requires to be exercised that no despotic over-riding of collateral and unexpected difficulties be attempted, rashly or indiscreetly, until these are met; as it need not be said that any proceedings taken by the Association or its representative board are much more likely to be successful by waiting till the line before it be pronounced all clear and safe, than by rushing on at express speed with some danger signal flaring in its face. Precipitate impulsive measures have nothing to recommend them. A prudent exercise of caution and determination will no doubt bye-and-bye make the Association 'a terror to evil-doers and a praise and protection to those that do well.' But while one of the main objects of the Association is to act for and to exercise a certain amount of jurisdiction over the profession—purposes which, when judiciously handled by means of its executive, namely, the Representative Board, become a vuluable and powerful element of control the British Dental Association, unlike other purely governmental bodies, has reserved to itself the privilege of establishing and maintaining the means of promoting friendly feeling as well as scientific intercourse among its members. It has done so by the establishment of a Benevolent Fund, by its great statutory and ever-acceptable periodic gatherings in different and distant centres of professional life throughout the country, and by the publication of a journal devoted to the literature of dentistry, and ever open to the community but serves to foster and facilitate the acquirements, the abilities, and the recording of the experience of every individual member composing it, according to the energy with which he may avail himself of the great and numerous advantages placed by it at his disposal.

It is, then, this great and beneficial association which I have now most cordially to welcome on the occasion of its meeting here in Scotland's capital—the city of Edinburgh. You are here as men pursuing and promoting the interests of science, faithfully forwarding the specialty to which your lives have been devoted. But I have to congratulate you, particularly the Southern strangers to our city, that such laudable pursuits have this year served a double purpose, and brought you to the 'stately Edinburgh thronged on crags.' I need not remind you of the romantic history, the features of picturesque attractiveness and natural grandeur, the monuments of antiquarian interest, the footmarks of a former age of splendor and renown, possessed by the city you are now honoring with your presenee. It is to no mimic scene to which you are introduced, no mere panoramic exhibition in which you now will walk. The actual scenes of old romantic legends and old associations will surround your steps on every side. A checqured history, broken as the memory of dreams, hangs over every shrine to which you may do homage. From the castle's rugged battlements and hoary towers, where kings were born and nobles pined and died, away down to deserted Holyrood, linked with the fate of Mary, fair but hapless Queen ot Scots, and Prince Charles Edward, names as enduring as the time worn 'Crags' and Lion Hill, keeping their weird watch and ward high over all, there is scarce a quaint or tottering edifice but has its own most wild or woeful history.

Having referred to the customary excursions being perhaps less necessary from Edinburgh than from any other city, Dr. Smith concluded:—A long array of great historic names, now low in the dust, once peopled the wynds and closes of that picturesque stretch of lofty habitations from where

The castle holds its state,
And all the steep slope down.
Whose ridgy back heaves to the sky,
Piled deep and massive, close and high,
Mine own romantic town.

These, I say, will, all in their own subordinate capacity and in the event of time permitting, lend an additional interest to your meeting here, and while such relics of a by-gone time suggest their own reflections, there is no want of more modern attractions in the legal, educational, eclesiastical, medical and charitable monuments of interest throughout the city to while away a leisure hour, and of which our law courts, our University, and our immense Infirmary may help to serve as types. And, now, gentlemen, offering you these remarks, I shall not detain you longer, nor further interpose between you and the more interesting business which lies before this meeting of the British Dental Association in Edinburgh.

Mr. Reid, L. D. S., Edinburgh, read a paper on "The Application of Dental Science in the Detection of Crime." Medical Jurisprudence occupied a wide field in the detection of crime in its varied forms, but in that part of it which might be termed dento-chirurgical jurisprudence it was narrowed down to the point of identification. While in several instances medico-legal evidence had been at fault, and a verdict of mistaken identity followed a lengthened and carefully-conducted trial, he had failed in finding on record an instance where the proof of identity brought out by the aid of dental science had been set at naught.

Mr. Henry Sewill, M. R. C. S., L. D. S., England, read a paper on "The Prevention of Caries." He said that caries was impossible without acid, and without vitiation of the secretions of the mouth. Without due exercise in mastication teeth could not easily be kept clean, and where the fuuction was impeded by the presence of tender teeth, these should be brought into a healthy state or extracted

Crowding of the teeth greatly prevented the constant beneficial rubbing and polishing of them, prevented the ready access of alkaline saliva to those surfaces where acid-forming substances find their location. He was afraid they could not hope that for the sake of the teeth of posterity men would be advised to pick out big-jawed wives, but they could at least seriousiy impress hygienists with the fact that the human jaw needed adequate use for its due development, and that no dietary could be perfect which was composed of uniformly bland and soft substances, calling for little or no mastication.

Mr. W. Campbell, L. D. S., England, Dundee, read the next paper on " Fractures of the Inferior Maxilla," illustrated by diagrams shown by the oxy-hydrogen light, and gave a successful demonstration of a new splint which he had devised and used in some cases in the Dundee Infirmary in treating fractures of the lower jaw.

Mr. G. W. Watson, L. D., Edinburgh, read a paper on "Antral Abscess." He enumerated the common causes, and the treatment he adopted, which was removal of diseased tooth and roots, washing out antrum with warm water, and afterwards with weak solution of permanganate of potash.

Dr. T. Stack thought the great majority of fluid diseases of the antrum arose from diseased teeth, and that though syringing with tepid water was good, usually some medicinal agent sholud be added to it.

Dr. Walker, M. R. C. S., L. D. S., England, read a paper on " Educational Centres." It was a notable fact that the population of Germany was more highly educated than that of the British Isles. Was this fact always to remain a standing blot in the history of our country ? If not, what means could be adopted to make it otherwise ? The British Dental Associatian might be one of the means to correct this general stagnation by considering the character of the primary and general education of their sons, and the result of public education in contradistinction of private.

Mr. J. R. Brownlie, L. D. S., Glasgow, read the last paper on the "Prevention of Irregularity." He said that while a regular arrangement of the teeth as a result of nature's handiwork was common enough, dentists were frequently called to see the results brought about by certain other forces acting at variance with those named, giving to the teeth a crowded arrangement which was a blemish in the mouth of beauty, and which was an important factor in the production of dental caries. The age at which prevention could be carried out was a very early one, a much earlier age than dentists usually had the opportunity of applying their knowledge. One of the earliest habits a child acquired giving rise to the deformity was that of sucking the thumb. Closely allied to this was the improper use of the feeding bottle. Next there was the possibility of the teeth of the first set becoming a cause of irregularity to those of the second. He also took occasion to refer to the practice of people setting no money value upon the services rendered and time occupied in consultations. An operation, he said, in itself so simple that the very veriest clown could hardly blunder, was credited with a money value, and the fee readily paid, while the special knowledge which had to be acquired by years of study, and which alone enabled one to deal intelligently with the conditions of the mouth, was set at naught too frequently as a thing not worthy of any recognition. Indefensible throughout, the practice was one which was essentially objectionable in regard to the case of children's mouths. If a dentist knew beforehand that on his decision would rest the question of fee or no fee, it was too much to expect from human nature that his decision would be free from bias, and founded purely on the merits of the case. The temptation was too great. If it were a question of the extraction of a temporary tooth, the operator should more than likely do what was wanted done by the parent, who was quite incapable of forming any inadequate opinion of its ultimate effect upon the mouth. Was it possible that under such conditions the opinion could be founded purely on the merits of the case? The position could not be defended, and there were but few of those parents or friends who interested themselves in the subject who were not capable of recognizing the risk to which the interests of the child were exposed, and with whom a word or two of explanation was all that was needed to secure to the operator unreservedly an opportunity to act or abstain from action *secundum artem.*

Mr. Balkwill urged that the teeth determined the position and form of the alveolus, and advocated the retention of the temporary canine as long as possible. He desired statistics of effects of premature removal of temporary teeth, and related a case in which the second deciduous molar was extracted and he did not anticipate evil results.

Dr. Williamson remarked that thumb-sucking affected only the front part of the alveolar arch, and also mentioned lip sucking as an item not mentioned by Mr. Brownlie. He alluded to an instance of the latter habit in which considerable hypertrophy of the lip ensued, and which eventually had to be removed.

Mr. Watson directed attention to two cases in Museum of the Odonto-Chirurgical Society, in each of which the deformity was caused by thumb-sucking.

Mr. Campbell mentioned two cases in which an upper and a lower bicuspid were retarded in a boy and a girl, æt. 13. By pressure upon the gum over the absent teeth they very shortly afterwards appeared.

Dr. Stack urged the retention of the temporary teeth, especially of the lower canine. He also remarked that if dentists put down their foot firmly, and said there shall be no consultations gratis, then, and not till then, would the practice complained of be given up.

Dr. Cunningham said that the public must be taught to know that it was for time and skill and medical advice, and not for a mere mechanical operation, that dentists were paid their fees.

A meeting of the subscribers to the Benevolent Fund was also held; and in the evening the members dined at the Waterloo Hotel, the Lord Provost being among the guests.
—*London Dental Record.*

